# Proton Beam Therapy Versus Photon Radiotherapy for Pediatric Neuroblastoma: A Systematic Review and Meta‐Analysis (TRP‐2025 Neuroblastoma)

**DOI:** 10.1002/cam4.71701

**Published:** 2026-03-09

**Authors:** Hazuki Nitta, Masashi Mizumoto, Kazushi Maruo, Yoshiko Oshiro, Yinuo Li, Masako Inaba, Takashi Saito, Sho Hosaka, Takashi Iizumi, Hiroko Fukushima, Ryoko Suzuki, Shosei Shimizu, Kei Nakai, Hideyuki Sakurai

**Affiliations:** ^1^ Department of Radiation Oncology University of Tsukuba Ibaraki Japan; ^2^ Department of Biostatistics, Institute of Medicine University of Tsukuba Ibaraki Japan; ^3^ Department of Radiation Oncology Tsukuba Medical Center Hospital Ibaraki Japan; ^4^ Department of Pediatrics University of Tsukuba Hospital Ibaraki Japan; ^5^ Department of Child Health, Institute of Medicine University of Tsukuba Ibaraki Japan; ^6^ Department of Pediatric Radiation Therapy Center/Pediatric Proton Beam Therapy Center Hebei Yizhou Cancer Hospital China

**Keywords:** meta‐analysis, outcomes, pediatric neuroblastoma, photon radiotherapy, proton beam therapy

## Abstract

**Background/Objectives:**

Radiotherapy (RT) is a standard component of treatment for high‐risk pediatric neuroblastoma. Proton beam therapy (PBT) offers dosimetric advantages over photon radiotherapy (XRT), but comparative clinical data remain limited.

**Methods:**

As part of the review project at our facility, we conducted a systematic review and meta‐analysis comparing survival outcomes between PBT and XRT. PubMed was searched for studies published between 1990 and 2022. Eligible studies included those for pediatric patients with neuroblastoma receiving curative‐intent RT and reporting overall survival (OS) or progression‐free survival (PFS). A random‐effects meta‐analysis and meta‐regression were performed.

**Results:**

Eighteen studies (6 PBT, 12 XRT) were included. The 1‐ and 2‐year OS rates were 97.2% and 92.0% for PBT, and 89.1% and 81.3% for XRT. Corresponding PFS rates were 96.1% and 86.1% for PBT, and 78.6% and 64.5% for XRT. Meta‐regression identified treatment modality as a significant factor for 2‐year OS and PFS. However, variability in patient background and treatment protocols may have influenced the results. Limited toxicity data suggested low rates of grade ≥ 3 acute adverse events in both groups.

**Conclusions:**

PBT gave survival outcomes that were at least comparable to XRT in pediatric neuroblastoma. Given its potential to reduce long‐term toxicity, PBT may be a reasonable alternative in selected cases. Further prospective studies are needed to validate these findings.

**Abbreviations**
LC: Local control
OS: Overall survival
PBT: Proton beam therapy
PFS: Progression‐free survival
XRT: Photon radiotherapy

## 1 Introduction

Neuroblastoma is one of the most common pediatric malignancies, after leukemia, brain tumors and lymphomas [1]. It is a solid tumor originating from undifferentiated neural crest cells [2, 3]. Neuroblastoma typically occurs in infants and young children under the age of 1–2 years, but exhibits highly heterogeneous clinical behavior. Some cases undergo spontaneous regression and have a favorable prognosis, while others are aggressive and refractory to treatment, and are often associated with MYCN gene amplification and poor outcomes [2, 3].

Due to this heterogeneity, cases are stratified as low‐, intermediate‐, and high‐risk based on factors such as age at diagnosis, clinical stage, and tumor biology, including MYCN status, histology, and chromosomal aberrations [4, 5, 6]. Treatment strategies vary accordingly. Observation or surgery alone is typically sufficient for low‐risk neuroblastoma, while intermediate‐risk cases are treated with a combination of surgery, chemotherapy, and occasionally radiotherapy. High‐risk neuroblastoma requires multimodal treatment, including intensive chemotherapy, surgical resection, high‐dose chemotherapy with autologous stem cell rescue, radiotherapy, and therapies such as differentiation and immunotherapy [7, 8]. Radiotherapy (RT) plays a key role in local control of both primary and metastatic lesions in high‐risk neuroblastoma [9], and is also used for palliation in emergencies such as respiratory distress due to liver metastases or spinal cord compression [10].

Photon radiotherapy (XRT) has commonly been used in this setting. However, the physical characteristics of XRT make it difficult to spare surrounding healthy tissues, which is a particular concern in pediatric patients, as it may lead to late effects such as growth impairment, endocrine dysfunction, and secondary malignancies [11, 12, 13]. Proton beam therapy (PBT) has recently garnered attention due to its superior dose distribution, enabling high‐dose delivery to tumors while sparing adjacent normal tissues [14]. These properties may reduce long‐term toxicities and improve post‐treatment quality of life. In Japan, PBT for pediatric cancers has been covered by public insurance since 2016, facilitating broader clinical use.

Neuroblastoma is a rare disease, and large‐scale randomized controlled trials (RCTs) directly comparing PBT and XRT are difficult to conduct. Therefore, systematic reviews and meta‐analyses are required to compare the efficacy and safety of these modalities. The review project at our facility was established to derive robust conclusions from limited data sources through rigorous evidence synthesis [15, 16]. In this context, the aim of this study is to assess whether PBT gives treatment outcomes that are at least non‐inferior to those of XRT in pediatric neuroblastoma by conducting a systematic review and meta‐analysis.

## 2 Materials and Methods

### 2.1 Selection Criteria for Meta‐Analysis

This study was conducted in accordance with the Preferred Reporting Items for Systematic Reviews and Meta‐Analyses (PRISMA) guidelines [17]. All retrieved articles were independently screened by two reviewers, and any discrepancies were resolved through discussion and consensus. To be eligible for inclusion in the meta‐analysis, studies had to meet the following criteria: patients with a clinical diagnosis of neuroblastoma; XRT or PBT administered with curative intent to primary or regional tumor sites; and radiotherapy primarily for the purpose of local control. Studies were also required to provide extractable data on overall survival (OS) and/or progression‐free survival (PFS). To ensure sufficient statistical robustness, we further limited inclusion to studies that enrolled at least 50 patients in the XRT group or at least 10 patients in the PBT group.

### 2.2 Search Strategy and Study Selection

A comprehensive literature search was conducted in the PubMed database for studies published between January 1990 and December 2022. The search terms used were: “neuroblastoma” AND (“radiotherapy” OR “proton”) AND (“children” OR “pediatrics”). A total of 1093 articles were identified. Two independent reviewers screened the titles and abstracts, and selected 350 articles deemed potentially relevant to radiotherapy for neuroblastoma. Full‐text articles were then reviewed in detail, and English‐language studies that clearly reported treatment outcomes and met the predefined inclusion criteria were included. To ensure a fair comparison, only XRT studies published from 2010 onward were considered, as most PBT studies were published after 2012.

**FIGURE 1:**
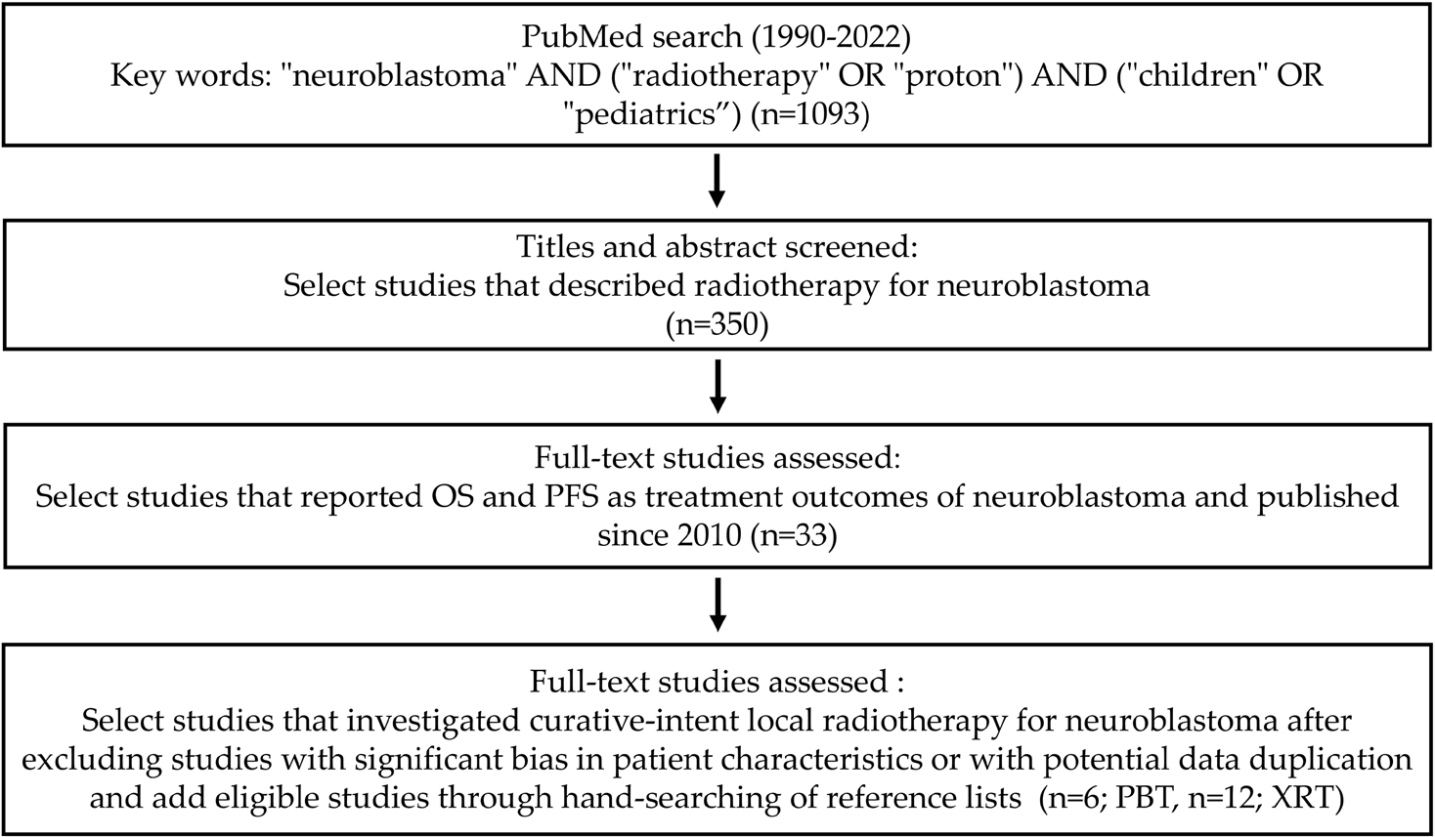
Flowchart of study selection. The flowchart illustrates the systematic selection of studies for the meta‐analysis.

Following this process, 8 studies using PBT and 25 studies using XRT were initially selected. The focus was then narrowed to studies that investigated curative‐intent local radiotherapy. Studies with significant bias in patient characteristics, such as those in which most cases were recurrent or with potential data duplication, were excluded. Additional eligible studies were identified through hand‐searching of reference lists. Ultimately, 6 PBT studies [18, 19, 20, 21, 22, 23] and 12 XRT studies [24, 25, 26, 27, 28, 29, 30, 31, 32, 33, 34, 35] met the inclusion criteria and were included in the meta‐analysis. The study selection process is illustrated in Figure 1.

### 2.3 Data Extraction

From each eligible study, data were extracted for first author's name, year of publication, country, study design, number of patients, number of deaths, number of local recurrences, 1‐, 2‐, and 3‐year OS, PFS, and local control (LC) rates; treatment modality (XRT or PBT); sex; median age or age range; follow‐up period; risk classification; presence of metastases; inclusion of recurrent cases; MYCN gene amplification rate; total radiation dose; and number of fractions. When 1‐, 2‐, or 3‐year OS, PFS, or LC rates were not explicitly reported in the text, values were estimated from Kaplan–Meier survival curves provided in the articles.

### 2.4 Statistical Analysis

Random‐effects meta‐analyses of 1‐, 2‐, and 3‐year OS and PFS were conducted separately for each treatment modality. The results are presented as forest plots. For studies with incomplete accuracy data, missing values were imputed based on the number of cases, the risk set size at each time point, and the mean dropout rate [36]. Heterogeneity was assessed using the I^2^ statistic. Random‐effects meta‐regression analyses were performed for each outcome, with treatment modality as the explanatory variable. All statistical analyses were conducted using R (R Core Team, Vienna, Austria) and the meta package [37]. A *p*‐value < 0.05 was considered to be statistically significant. Due to the limited reporting of 1‐, 2‐, and 3‐year LC rates in the included studies, a formal comparative analysis of LC between XRT and PBT could not be performed.

## 3 Results

The patient characteristics by treatment modality (XRT vs. PBT) were as follows: median age, 37.3 vs. 41.4 months; male sex, 57.9% vs. 52.8%; median follow‐up period, 55.5 vs. 41.5 months; high‐risk classification, 100% vs. 88.8%; distant metastases, 89.8% vs. 79.0%; and MYCN amplification, 39.8% vs. 38.0%. The baseline characteristics by treatment modality are presented in Table 1.

**TABLE 1:** Backgrounds of patients treated with XRT and PBT.

Factor	XRT	PBT
Total patients	3265	158
Age		
Median (months)	37.3	41.4
Range	24–54	36–48
Male		
Median (%)	57.9	52.8
Range	50–63.2	42.6–77.8
Median follow‐up period		
Median (months)	55.5	41.5
Range	24–119.6	14.9–60.2
High‐risk classification		
Median (%)	100	88.8
Range	100–100	78.6–100
Distant metastases		
Median (%)	89.8	79
Range	80.5–100	35.7–94.4
MYCN amplification		
Median (%)	39.8	38
Range	16–48.1	32–65.9

Abbreviations: PBT, proton beam therapy; XRT, photon radiotherapy.

**FIGURE 2:**
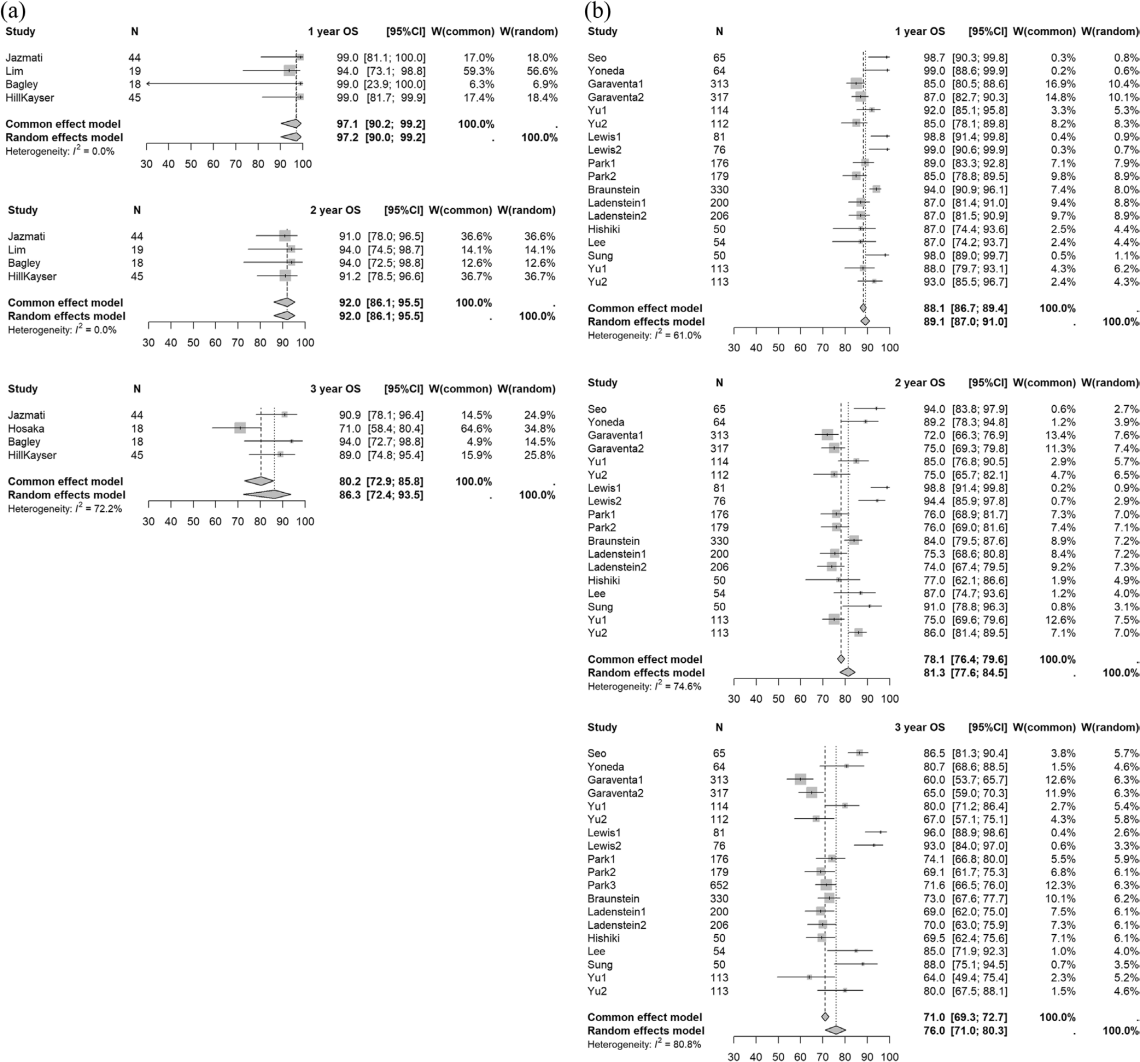
Forest plot of 1‐ to 3‐year OS rates for (a) PBT and (b) XRT. The forest plot displays the 1‐ to 3‐year OS rates for studies for neuroblastoma. Each data point represents an individual study, with the corresponding CI shown as horizontal lines. The pooled estimates of OS rates are displayed, providing a summary of the survival outcomes across different time points. Abbreviations: OS, overall survival; PBT, proton beam therapy; XRT, photon radiotherapy; CI, confidence interval.

In the meta‐analysis of the 18 selected studies, the 1‐, 2‐, and 3‐year OS rates for XRT vs. PBT were 89.1% (95% CI: 87.0–91.0) vs. 97.2% (90.0–99.2) (*p* = 0.0371), 81.3% (77.6–84.5) vs. 92.0% (86.1–95.5) (*p* = 0.0104), and 76.0% (71.0–80.3) vs. 86.3% (72.4–93.5) (*p* = 0.1210), respectively. Forest plots of 1‐, 2‐, and 3‐year OS by treatment modality are presented in Figure 2.

**FIGURE 3:**
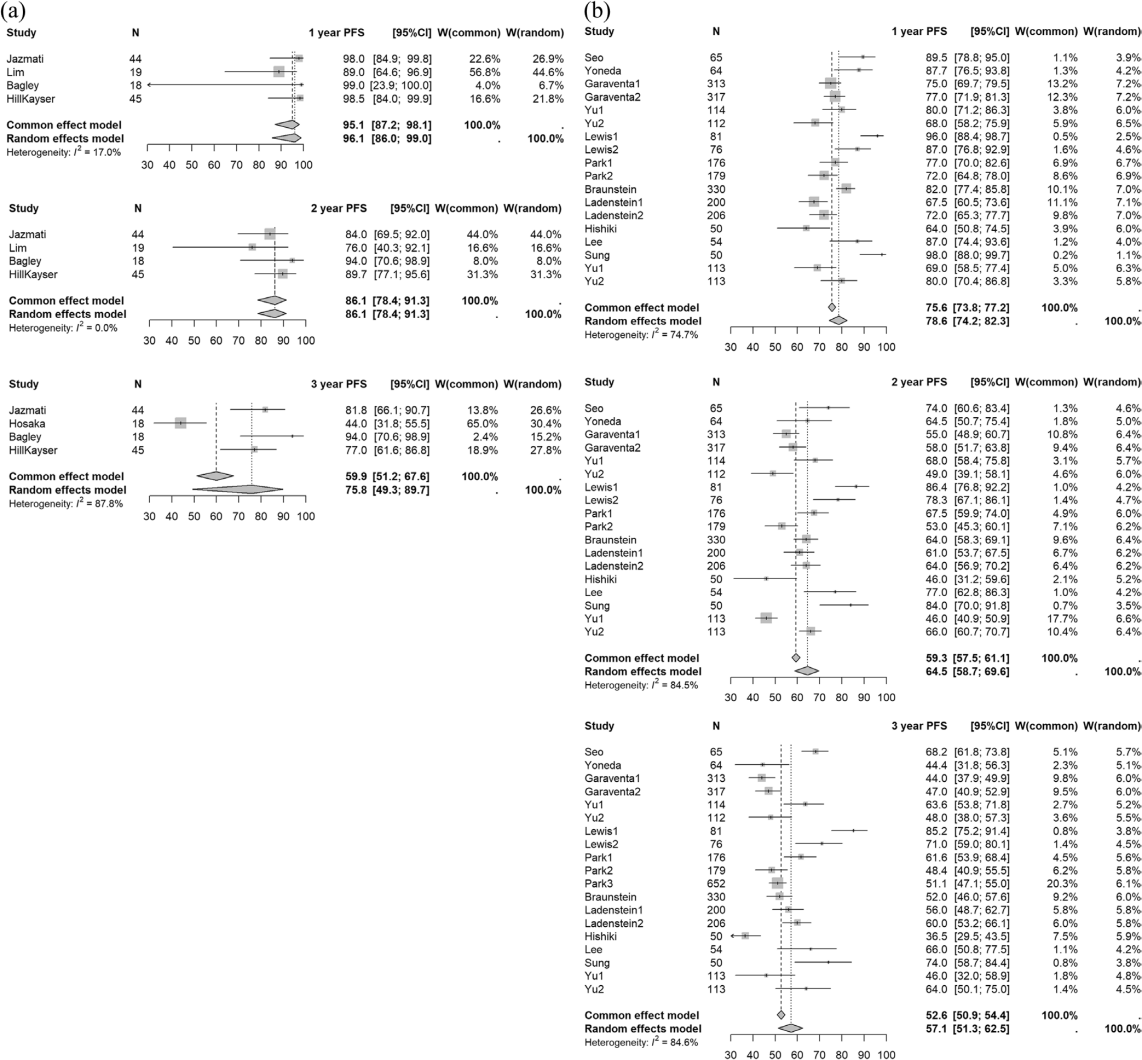
Forest plot of 1‐ to 3‐year PFS rates for (a) PBT and (b) XRT. The forest plot displays the 1‐ to 3‐year PFS rates for studies for neuroblastoma. Each data point represents an individual study, with horizontal lines indicating the corresponding CI. The pooled estimates provide a summary of PFS outcomes over different time intervals. Abbreviations: PFS, progression‐free survival; PBT, proton beam therapy; XRT, photon radiotherapy; CI, confidence interval.

The 1‐, 2‐, and 3‐year PFS rates for XRT vs. PBT were 78.6% (95% CI: 74.2–82.3) vs. 96.1% (86.0–99.0) (*p* = 0.0041), 64.5% (58.7–69.6) vs. 86.1% (78.4–91.3) (*p* = 0.0011), and 57.1% (51.3–62.5) vs. 75.8% (49.3–89.7) (*p* = 0.0928), respectively. Forest plots of 1‐ to 3‐year PFS by treatment modality are presented in Figure 3. Due to the limited availability of extractable data, 1‐year LC rates could not be calculated for the XRT group. Among the PBT studies, 1‐year LC rates ranged from 92% to 100%, with a median of 100%.

A meta‐regression analysis was performed using available data from each study, with treatment modality (XRT vs. PBT), presence of distant metastasis (yes vs. no), and MYCN status (positive vs. negative) as covariates. The analysis revealed that PBT was significantly associated with improved 2‐year OS and 1‐ and 2‐year PFS. In contrast, distant metastasis and MYCN amplification were not significantly associated with treatment outcomes. Details of the meta‐regression analysis are provided in Table 2.

**TABLE 2:** Meta‐regressions of potential predictive factors for 1‐ to 3‐year overall survival and 1‐ to 3‐year progression‐free survival.

Factors	estimate	se	zval	*p*val	ci.lb	ci.ub
1‐year OS						
Treatment modality	0.9452	0.7855	1.2034	0.2288	−0.5943	2.4847
Distant metastasis	0.0194	0.0244	0.7932	0.4277	−0.0285	0.0672
MYCN status	0.0048	0.0157	0.3077	0.7583	−0.0259	0.0355
2‐year OS						
Treatment modality	1.0540	0.5265	2.0019	0.0453	0.0221	2.0859
Distant metastasis	−0.0060	0.0213	−0.2833	0.7769	−0.0477	0.0357
MYCN status	0.0043	0.0166	0.2593	0.7954	−0.0282	0.0368
3‐year OS						
Treatment modality	0.4086	0.4281	0.9545	0.3398	−0.4304	1.2477
Distant metastasis	−0.0003	0.0220	−0.0118	0.9906	−0.0435	0.0430
MYCN status	−0.0075	0.0179	−0.4187	0.6754	−0.0425	0.0275
1‐year PFS						
Treatment modality	1.7290	0.7246	2.3860	0.0170	0.3087	3.1492
Distant metastasis	−0.0335	0.0258	−1.2959	0.1950	−0.0842	0.0172
MYCN status	−0.0061	0.0167	−0.3635	0.7162	−0.0388	0.0267
2‐year PFS						
Treatment modality	1.4889	0.4604	3.2342	0.0012	0.5866	2.3911
Distant metastasis	−0.0271	0.0186	−1.4520	0.1465	−0.0636	0.0095
MYCN status	−0.0125	0.0133	−0.9467	0.3438	−0.0385	0.0134
3‐year PFS						
Treatment modality	0.3338	0.3532	0.9450	0.3447	−0.3585	1.0261
Distant metastasis	0.0077	0.0195	0.3958	0.6922	−0.0305	0.0459
MYCN status	−0.0083	0.0148	−0.5595	0.5759	−0.0372	0.0207

Abbreviations: OS, overall survival; PFS, progression‐free survival.

## 4 Discussion

Radiation therapy is a well‐established component of multimodal treatment for high‐risk neuroblastoma. Although no RCTs have definitively evaluated its efficacy, RT is primarily used to achieve local tumor control [9, 38]. Clinical series have consistently supported its utility; for instance, Bradfield et al. reported local control in 16 of 17 high‐risk cases following RT of 21 Gy [9], and Gatcombe et al. obtained a 3‐year LC rate of 94% with a RT dose of 21–24 Gy [38].

While XRT has long been a standard method, advances in technology have expanded interest in PBT. The Bragg peak enables PBT to deliver a maximal dose to the tumor target while sparing normal tissues beyond the target [39]. This physical advantage results in reduced radiation exposure to surrounding organs‐at‐risk (OARs), even compared with advanced X‐ray techniques such as intensity‐modulated radiation therapy (IMRT). Hill‐Kayser et al. and Hattangadi et al. have shown that PBT achieves significant reductions in doses to critical organs, including the liver, kidneys, lungs, and heart [39, 40]. This is particularly relevant in pediatric neuroblastoma, which affects very young children who are vulnerable to long‐term effects of RT, including secondary malignant neoplasms (SMNs) and musculoskeletal complications [41, 42, 43, 44]. Zhen et al. found a 4.36‐fold increased SMN risk among neuroblastoma survivors [41] and Ducassou et al. showed the dose‐dependence of late musculoskeletal effects [42]. Ferris et al. also highlighted the importance of balancing efficacy and toxicity in this patient population [45]. These findings support the rationale for minimizing unnecessary doses to normal tissues, which is a key advantage of PBT.

Although individual studies of neuroblastoma have reported favorable outcomes with PBT, direct comparisons with XRT are limited by the rarity of the disease. Our meta‐analysis synthesized available evidence to compare survival outcomes between PBT and XRT. The results indicate that PBT demonstrated survival outcomes that were in some cases more favorable than those of XRT, particularly for 1‐ and 2‐year overall and progression‐free survival. However, as the PBT group included a slightly lower proportion of high‐risk patients and exhibited some variability in patient background, these differences should be interpreted with caution. While LC could not be meta‐analyzed due to limited data, studies such as that by Hill‐Kayser et al. suggest favorable control rates with PBT [19].

The studies from which our PBT data were drawn report low rates of grade ≥ 3 acute toxicity, consistent with dosimetric advantages. However, comprehensive assessment of late effects and SMN risk remains limited due to short follow‐up periods. As noted by Ducassou et al. and others [42, 46, 47, 48], late effects may emerge years after treatment and are often dose‐related. Interestingly, Ferris et al. found no added benefit of dose escalation beyond 21.6 Gy in terms of LC or survival, even in cases with gross residual disease or positive margins [45]. This suggests that a key benefit of PBT lies not in dose intensification, but in its ability to spare OARs.

Among late effects, particular attention should be paid to vertebral irradiation in pediatric patients, as asymmetric or inhomogeneous dose distributions across growing vertebral bodies may increase the risk of long‐term spinal deformities, including scoliosis and vertebral hypoplasia, as noted by Ducassou et al. and others [42, 46, 47, 48]. In line with this concern, recent consensus recommendations from the SIOP Europe radiotherapy working group emphasize the importance of maintaining vertebral dose homogeneity whenever feasible, especially in younger children [49]. Although robust clinical outcome data such as LC or PFS directly related to vertebral dose homogeneity remain limited due to the relatively recent recognition of this issue, recent dosimetric analyses have demonstrated that careful treatment planning is required to achieve adequate vertebral dose homogeneity, particularly in proton therapy, where steep dose gradients and robust CTV concepts may otherwise result in unintended vertebral dose asymmetry [50].

This study has several limitations inherent to meta‐analyses of observational studies, including heterogeneity in patient populations, treatment protocols, PBT techniques (e.g., 3D‐CPT vs. IMPT), follow‐up periods, and the relatively small number of eligible studies. Our inclusion criteria were specifically designed to prioritize robust oncological endpoints in order to provide clinically meaningful evidence. Although this selective approach restricted the number of eligible studies, it was essential to enhance the methodological robustness and the reliability of the synthesized results. Furthermore, the limited number of PBT studies reflects the current scarcity of high‐quality clinical evidence and should therefore be interpreted as an inherent limitation of the present meta‐analysis. Another critical consideration is that the clinical outcomes of high‐risk neuroblastoma have improved dramatically in recent decades, driven by the intensification of multimodal therapies. Although we endeavored to synchronize the treatment eras of the XRT and PBT group during the literature selection process, the PBT group inherently consist primarily of contemporary cases. Consequently, the possibility of confounding bias arising from disparities in systemic treatment regimens cannot be entirely excluded when comparing outcomes with the XRT group. Nevertheless, based on the limited evidence available, this study suggests that PBT achieves survival outcomes that are at least comparable to those of XRT, with potential advantages in selected patients. Future studies should aim to compare PBT and advanced photon techniques, such as IMRT, within the framework of identical systemic treatment protocols.

## 5 Conclusions

In conclusion, RT remains essential for high‐risk neuroblastoma, and PBT offers dosimetric advantages that may translate into reduced toxicity while maintaining effective tumor control. This study suggests that PBT offers survival outcomes that are generally comparable to those of XRT in pediatric neuroblastoma, with some analyses indicating potentially improved short‐ to mid‐term survival. To establish more robust evidence, prospective comparative studies with standardized treatment protocols and well‐aligned patient backgrounds are warranted.

## Author Contributions

Conception/design: M.M., and Y.O., Collection and/or assembly of data: Y.L., M.M., Y.O., M.I., T.S., S.H., T.I., H.F., R.S., H.N., S.S., and K.N., Data analysis and interpretation: M.M., and K.M., Manuscript writing: H.N., Final approval of manuscript: H.S. All authors made substantial contributions to the study concept or the data analysis or interpretation; drafted the manuscript or revised it critically for important intellectual content; approved the final version of the manuscript to be published; and agreed to be accountable for all aspects of the work.

## Funding

This work was supported by JSPS KAKENHI Grant Number JP22K07764.

## Ethics Statement

The authors have nothing to report.

## Conflicts of Interest

The authors declare no conflicts of interest.

## Data Availability

The data that support the findings of this study are available from the corresponding author upon reasonable request.
